# In Search for Symbolic Qualities of Iron: The Metal of Life

**DOI:** 10.3389/fphar.2016.00220

**Published:** 2016-08-03

**Authors:** Zvi Ioav Cabantchik

**Affiliations:** Department of Biological Chemistry, Alexander Silberman Institute of Life Sciences, The Hebrew University of JerusalemJerusalem, Israel

**Keywords:** iron, natural medicine, plants, medicinal, anemia, therapeutics

“The past is never dead. It's not even past” (William Faulkner)

Iron, classically known as the Metal of Mars, has been universally perceived as the symbol of human strength, obstinacy, fortitude, honor, courage, sharpness (of body and mind), tenacity, and confidence in power. Legendary personalities like the knight Götz von Berlichingen (the one with prosthetic iron hands) or Margaret Thatcher (the iron lady) epitomized the metal properties in human character. However, it was in Rudyard Kipling's poem “Cold Iron” that a pugnacious Baron elevates iron to a supreme level by proclaiming “cold iron as master of them all” (leaving gold for the mistress and silver for the maid). Ironically, the Baron's strong belief in iron supremacy leads him to wage war against the King, but ends up defeated and humiliated. Although pardoned despite of betraying the King, he rather adheres to his original “iron master” creed to be sentenced with “iron out of Calvary is master of them all.” It is a question of interpretation whether or not the poem conveys a theological message or a metaphor for a feigned feeling of supremacy, its recurrent use by “siderophiles” as a symbol of iron's preponderance in living organisms is perplexing (Beutler, 2002; Sheftel et al., 2012). Shouldn't a more benevolent “master iron” icon be adopted to represent iron as the metal of life?

We learn from history that a symbolism based on genuine iron master qualities was proposed more than a quarter of a millennium ago (Rodríguez Marín, 1925; Lasso de la Vega y Cortezo, 1988; Beutler, 2002; Sheftel et al., 2012; Beecher, 2015; Olmedilla y Puig, 2015). It was in 1574, in the town of Seville, the gateway to the recently discovered and conquered New World, where El Señor Doctor Nicolás Monardes (1493/1508?-1588) made public an essay entitled “Dialogue about the grandeur of iron” (which excels over other metals and is in highest demand to human service and of great medicinal qualities) (Monardes, 1549/1574) (Figure 1). The essay appears as a separate section in the *Medicinal History of Plants Imported from the New World* published together with two (previously published) sections and a new appended one (Monardes, 1549/1574)^1^. The author is a graduate in Arts and Philosophy (and later in Medicine) from the famous Universidad de Alcalá de Henares, who excelled in the practice and writing of Medicine, Botany, and Alchemy. His contributions earned him honorary titles such as “Father of Spanish Pharmacology” and “Discoverer of Fluorescence.” His associations with colonial traders of medicinal plants and minerals were instrumental in his scientific career as a botanist and in his medical practice. However, commercial misfortunes with overseas traders led him to declare bankruptcy and hide for a decade in a monastery, before managing to repay his creditors and to openly resume his medical practice and scientific endeavors. Monardes collected, studied, cataloged, and grew in his own garden some rare plants from which he made extracts that were applied to patients. He practiced medicine using classical books as well as novel information gathered from the American natives as guidelines. On the basis of Galenic medical reasoning (enriched with books from Arabic and Jewish scholars) and his own clinical experience, he came out with a singular treatise of medicine that comprises botanical descriptions, pharmaceutical preparations, miraculous cures, and guidelines to promote the exploitation of overseas resources (Monardes, 1549/1574).

**Figure 1:**
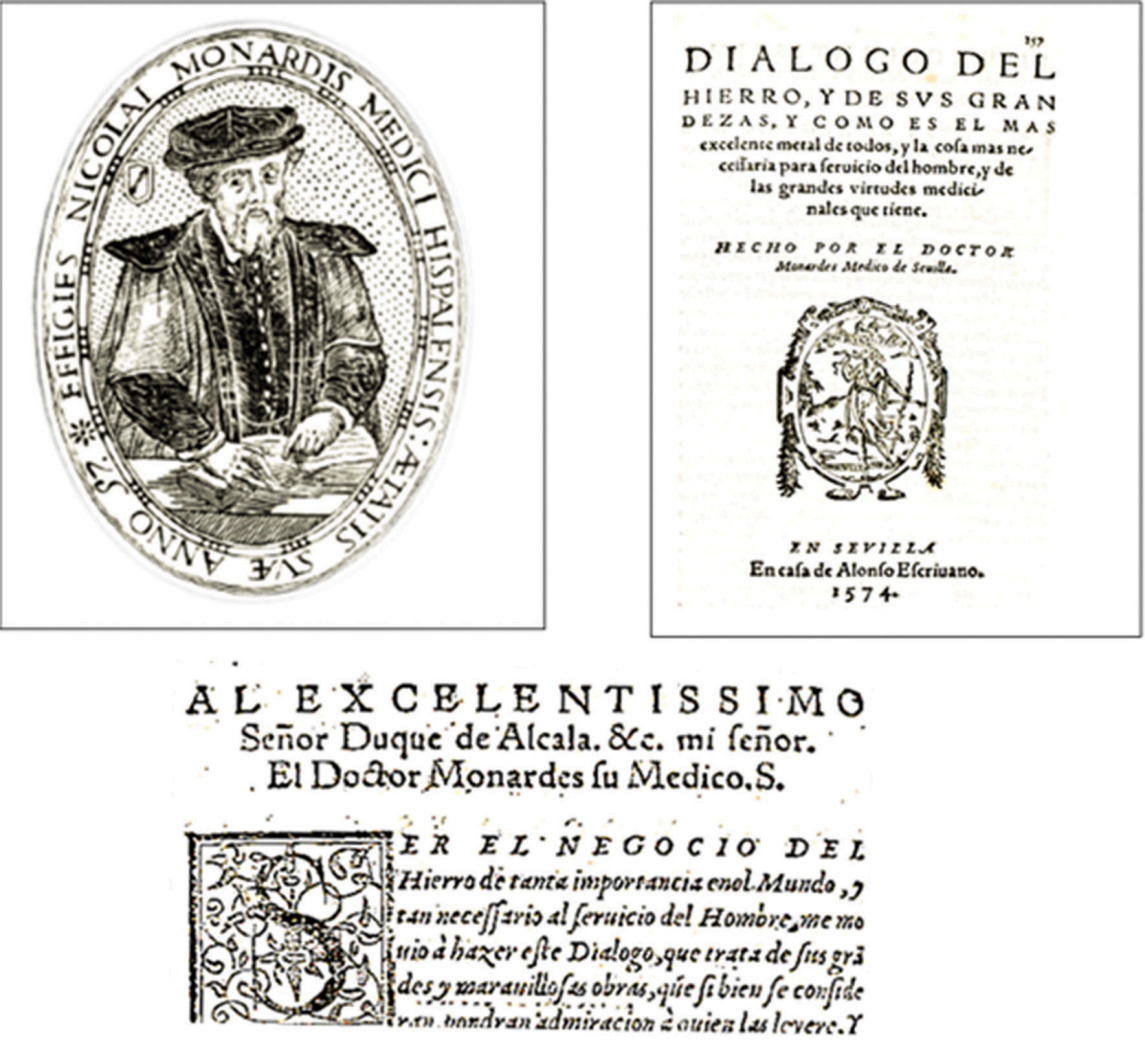
**Top left:** Portrait of Nicolás Monardes (Taken from “Elogio hecho por el Illust. S. Gonçalo Çati eco de Molina, Retrato del autor que se vee en su Museo”—Eulogy made by the illustrious Senior Gonçalo Çati, eco de Molina, Portrait of the author which can be seen in his Museum). **Top right:** Front page of the book “Dialogo del hierro…” Monardes, N. (1549/1574). (Dialogue of iron and its grandeur as it is the most excellent of all metals, and of best service to human and endowed with greatest medicinal virtues). Done by the Doctor Nicolás Monardes of Seville. The book was dedicated to the Duque de Alcalá, (Fernando Enríquez de Ribera y Portocarrero) and is wife (referred as & c. = compañera) who was no other but the Señora Juana Cortés de Zúñiga, daughter of Hernán Cortés, the conquistador of México. **Bottom:** First page of text Monardes, N. (1549/1574): TO HIS HIGHNESS, Sr Duke of Alcalá & c. my senior your physician Dr. Monardes, S., “BEING THE BUSINESS of iron OF such worldwide importance, and of such a service to Humans, I tried to compose this Dialogue, that deals with its great and wonderful uses, that if faithfully followed, will confer a great admiration to the readers.”

The Renaissance scholar followed a classical style of writing (Monardes, 1536, 1549/1574), using Plato's Dialogues as a model for asking questions (by Burgos, the “praxis” apothecarian-pharmacist), and Monardes (the “episteme-scientia” doctor) providing erudite answers assisted occasionally by Ortuño (the Basque “techne” blacksmith). He exposed iron's superior qualities over the much adored precious metals, gold and silver, which were brought from the Spanish colonies, thereby indicating that the real qualities should be assessed by the ability of the metal in question to provide both good health and living comfort furnished by instruments, utensils, and ornaments. He lead Burgos to the blacksmith's workshop, where Ortuño made a thorough exposure and demonstrated the various metallic qualities of iron according to its (geographic) site of extraction and its amenability to manufacture useful objects such as sewing needles and navigator compasses (both venerated by the Indians, who traded them for gold), house ornaments as well as weapons for conquering new lands or punishing sinners (!).

Using alchemical logic, Monardes described the composition of iron and its multiple uses for producing therapeutic miracles or making instruments used in surgery and barbershops (often by the same “professionals”). He gave credit to the old masters Plato, Hippocrates, Galenus, and others for their wisdom and guidance, but when appropriate, according to his own experience, he dared to dispute them^2^. He not only occasionally praised old miracles as credible (e.g., the shepherd Melampus that with magical powers cured infertility by giving acid wine aged over rust knife scrapings) but also emphasized the importance of using pure/washed iron (oxide) sources for preparing iron-based medications devoid of any traces of lethal elements (lead, copper, or vitreous -silicate). The medical virtues of iron-based medications are exemplified by their ability to treat bloody diarrhea, perianal fistulas, vaginal discharges, wounds, weakness, hemorrhoids, cystitis, styptis, or hemorrhage. Monardes was acquainted with classical and modern medical literature, meeting Burgo's, professional curiosity and inquisitive questions that probe the doctor's knowledge and wisdom *(“sabiduria*”). Burgo wanted to go on asking, “Wishing the sunset to be delayed, as there is so much to be said about iron that Medicine has forgotten, being no human disease from toe to head-hair that iron is not involved and he is privileged to be next to an erudite man that is so knowledgeable of most important things.”

Monardes books were translated to European languages and remained popular until the Eighteenth century. Many of the practices he advocated have been preserved both as home remedies and as prescribed medicines^3^.

His classification of old and newly discovered plants (from the colonies) has been recognized with some having found their way into present pharmacopeia, such as tobacco and sassafras and *Monarda punctata*, the source of thymol used as an intestinal antiseptic giving urine a greenish tint^4^.

Nicolás Monardes is the “*nueva persona”* that the Spanish Renaissance produced—an educated professional in Medicine who excelled, thanks to his inquisitive and highly entrepreneurial but also humanistic spirit. He is a unique historical personality who was fascinated by the properties of iron, long before it was scientifically recognized as the metal of life and preceding the industrial revolution. His passion was transmitted through books that disseminated medical information to professionals and to laymen the potential of natural remedies for comforting if not curing body and soul.

Monardes earns a most distinguished place in the Pantheon of biomedical research of metals.

## Author contributions

ZC conceived and wrote the manuscript.

## Author notes

Based on an invited lecture “The sounds of Iron” given as Masterclass Lecture at the European Iron Club meeting held in Innsbruck in April 2016, representing IBIS (International Bioiron Society).

### Conflict of interest statement

The author declares that the research was conducted in the absence of any commercial or financial relationships that could be construed as a potential conflict of interest.

## Footnotes

^1^In Spanish: “Historia medicinal de las cosas que se traen de nuestras Indias Occidentales que sierven en Medicina” translated by the English traveler John Framton and printed in London as “The Three Bookes written in the Spanishe tongue by the Famous Phisition Monardes, N. (1549/1574)” and reprinted in the same year under the title “Joyfull Newes out of the newe founde worlde.”

^2^Quid nobis certius ipsis sensibus esse potest, qui vera ac falsa notemus”. Lucretius “De rerum natura” (“What can be more certain than our own senses to distinguish between truth and falsehood.” Lucretius “The Nature of things”.

^3^Although iron salts have been used topically in Ancient Greek as styptics, namely to stop hemorrhage, they proved useful even today as Monsel's liquid or paste (active agent is basic ferric sulfate or iron subsulfate). It can also be useful after cryotherapy, punch biopsy and loop electrosurgical excision procedure or for covering bleeding areas on the cervix to stem the flow of blood.

^4^F.A. Oski opens his 1979 Am. J. Dis. Child (133: 315) classical paper on “Non hematological manifestations of Iron deficiency” with a series of questions about symptoms that are indicative of iron deficiency, and quotes Monardes as a pioneer in the field.

## References

[B1] Beecher D. (2015). Nicolás Monardes, John Frampton and the Medical Wonders of the New World, in Humanismo e Ciência: Antiguidade e Renascimento, eds Andrade A. M. L. Mora M. de Torrão C. Nunes J. M. (Aveiro: Universidade de Aveiro, Imprensa da Universidade de Coimbra), 141–160.

[B2] Beutler E. (2002). The history of iron in medicine. Blood Cells Mol. Dis. 29, 297–308. 10.1006/bcmd.2002.0560 12547220

[B3] Lasso de la Vega y Cortezo J. (1988). Biografia y Estudio Critico de Las Obras del Médico Nicolás Monardes. Sevilla: Padilla Libros.

[B4] Monardes N. (1549/1574). 1era, 2da, y 3 Cera Partes de la Historia Medicinal de las Cosas que se Traen de Nuestras Indias Occidentales que Sirven en Medicina, Sevilla, A. Escribano 1574: A. De Rosa et Partibus eius. De Succi Rosarum Temperatura, Nec Non de Rosis Persicis ca.1540; B. De Malis Citriis, Aurantis Aclimoni 1564; Tratado de la Piedra Bezaar, Sevilla, A.Escribano-1574; Diálogo del Hierro-1574; Tratado de la Nieve y del Beber Frío-. Sevilla: Padilla Libros.

[B5] Monardes N. (1536). Diálogo Llamado Pharmacodilosis. Sevilla: Padilla Libros.

[B6] Olmedilla y Puig J. (2015). Estudio Histórico de la Vida y Escritos de Un Sabio Médico Español Del Siglo XVI, Nicolás Monardes, Madrid, Impr,-Hijos de M.G. Hernández, 1897 Presented at the 2015. Aveiro Available online at: https://digitalis.uc.pt/handle/10316.2/35692

[B7] Rodríguez Marín F. (1925). La verdadera biografía del Doctor Nicolás Monardes. Revista de Archivos. Madrid (printed excerpts from a conference presented in the Ateneo de Madrid, December 4, 1913).

[B8] Sheftel A. D. Mason A. B. Ponka P. (2012). The long history of iron in the Universe and in health and disease. Biochim. Biophys. Acta 1820, 161–187. 10.1016/j.bbagen.2011.08.002 21856378 PMC3258305

